# A Laboratory Evaluation of Medicinal Herbs Used in China for the Treatment of Hand, Foot, and Mouth Disease

**DOI:** 10.1155/2013/504563

**Published:** 2013-03-10

**Authors:** Xiaoqing Chen, Chunyang Wang, Lanfang Xu, Xiaoshuang Chen, Wei Wang, Guang Yang, Ren Xiang Tan, Erguang Li, Yu Jin

**Affiliations:** ^1^Medical School and Jiangsu Key Laboratory of Molecular Medicine, Nanjing University, 22 Hankou Road, Nanjing 210093, China; ^2^State Key Laboratory of Pharmaceutical Biotechnology and School of Life Sciences, Nanjing University, 22 Hankou Road, Nanjing 210093, China; ^3^Rosalind Franklin University of Medicine and Science, North Chicago, IL 60064, USA; ^4^Nanjing Children's Hospital, Nanjing Medical University, 72 Guangzhou Road, Nanjing 210008, China

## Abstract

Enterovirus 71 (EV71) and coxsackievirus A16 (CVA16) are the causative agents of hand, foot, and mouth disease (HFMD). During recent epidemics of HFMD in China, medicinal herbals and preparations containing herbal extracts have demonstrated therapeutic efficacy with relative safety profiles. There have been no microbiological studies to validate their usefulness for HFMD. We selected 12 commonly used herbs for HFMD from government recommended guidelines as well as published reports and tested for their antiviral activity and anti-inflammatory activity. A water extract of *Houttuynia cordata* Thunb. (HCT) inhibited EV71 infection significantly and was marginally active against CVA16 infection. The IC_50_ (concentration to have 50% inhibitory effect) values of HCT against a Fuyang strain and a BrCr strain of EV71 were determined at 8.9 **μ**g/mL and 20.6 **μ**g/mL, respectively. *Mentha haplocalyx* Briq. (MHB) water extract was active against CVA16, with an IC_50_ value of 70.3 **μ**g/mL. The extract did not exhibit activity against EV71 infection. Although the majority of the extracts showed no activity against viral infection, several extracts demonstrated activity in blocking proinflammatory response by viral infection. This study therefore validates the effectiveness of Chinese herbs for HFMD since some formulations containing the correct combination of the herbs can block viral replication as well as proinflammatory response of HFMD.

## 1. Introduction

Hand, foot, and mouth disease (HFMD) is a common viral illness that usually affects infants and children younger than 5 years old. Sporadic occurrence of the disease has been reported globally since first reported in New Zealand in 1957, but larger outbreaks with higher morbidity and mortality have increased recently in the Asian Pacific region, including an outbreak in Fuyang, China, that affected close to a half million children with 126 deaths in 2008 [1]. Coxsackievirus A16 (CVA16) and enterovirus 71 (EV71) are the causative agents for HFMD, accounting for over 70% of cases during the outbreak in China in 2008 [2]. The viruses are members of the Picornaviridae family and are spread through contact with virus-containing body fluids, respiratory droplets, and feces. There are no vaccine or antiviral drugs available for HFMD. Although immunoglobulin and antiviral agent ribavirin are commonly used, the efficacy remains uncertain [3, 4]. Good personal hygiene, including hand washing and disinfection of surfaces in child care facilities, remains therefore as the most effective approach to reduce the transmission rate of HFMD [5, 6].

During recent epidemics of HFMD in China, herbals and preparations containing herb extracts have demonstrated therapeutic efficacy against the disease. Some of the herbs or herbal preparations have been shown to ameliorate the symptoms and others to shorten the course of the disease [7]. There was no definitive description of symptoms related to HFMD in ancient manuscripts and publications in China until recent outbreaks of the disease. To deal with the clinical manifestations characterized with mild fever and rash, practitioners of Chinese medicine reasoned and have effectively treated HFMD as dampness and damp heat. We compiled a list of commonly used herbs with implication for HFMD therapy (Table 1) from a list of recommended remedies by government agencies in China and from published reports [7–9]. Most of the herbs in Table 1 have traditionally or folklorically been used for inflammatory or infective diseases. In addition, most of the herbs listed in Table 1 have not been investigated for their antiviral activity against pathogens of HFMD disease, although some of them have demonstrated antiviral activity. Less is known about the active components of those plants.

The objectives of this study were to validate the effectiveness of the herbs microbiologically by measuring their antiviral activity. In addition we also studied whether the herbs possessed anti-inflammatory activity since one of the symptoms of HFMD is an acute inflammatory response. Here we report the antiviral and anti-inflammatory activity evaluation of the commonly used herbs for HFMD therapy in China.

## 2. Materials and Methods

### 2.1. Literature Survey of Plant Materials, Plant Material Authentication, and Preparation of Hot Water Extracts

In an effort to identify most commonly used Chinese medicines for HFMD, Zhang et al. surveyed Chinese literatures from 1988 to 2009 and identified *Forsythia suspense, Lonicera japonica,* and *Glycyrrhiza* spp. as among the most commonly used herbs for HFMD [8]. Although some minerals like gypsum mineral (CaSO_4_ *·*2H_2_O) and talcum powder (magnesium silicate hydrate) or insect molting from cicada have been prescribed for HFMD, we focused our investigation on herbal medicines. We generated a list of the most frequently used plant materials for HFMD by combining those identified by Zhang and colleagues with those recommended by government agencies (Table 1).

On the basis of the frequency of their uses for HFMD, twelve medicinal materials were purchased from local herbal markets in Nanjing. Voucher specimens were stored at the Laboratory of Microbial Science, School of Medicine, Nanjing University. The corresponding Latin names, followed by abbreviations of the names and parts of the plants used for pharmaceutical and clinical practices, are listed in Table 1.

We used a modified protocol to prepare water extracts [10]. In brief, two grams of ground specimen was soaked with 30 mL of distilled water at room temperature (RT) for an hour with occasional shaking. Subsequently, the material was warmed up to 65°C in a water bath and extracted for 4 hr. The material was extracted one more time, and the pooled liquid fractions were left at RT for overnight to let insoluble materials settle down. Finally, water soluble materials were separated from the insoluble debris by centrifugation (5000 ×g, 15 min). The major chemical components in those extracts were validated following protocols given in Chinese Pharmacopeia (2010 ed.). Those showed that antiviral activities were further studied to validate the presence of characteristic chemicals in the plants following some literatures (e.g., used HPLC profiling for *H. cordata* and *M. haplocalyx*). The water extracts were then dried by lyophilization. The lyophilized powders were stored at −70°C and dissolved in distilled water to make stocks of 20 mg/mL before experiments.

The study design and assays used to validate the activity of the selected herbs are summarized in Figure 7.

### 2.2. Cells and Viruses

The rhabdomyosarcoma (RD) cell line and African green monkey kidney epithelial (Vero) cell line were purchased from Cell Bank of Chinese Academy Sciences (Shanghai, China). The cells were maintained in Dulbecco's modified Eagle's medium (DMEM) with high glucose, supplemented with 10% heat inactivated fetal bovine serum, 2 mM L-glutamine, nonessential amino acids, and sodium pyruvate (the medium and supplements are from Life Technologies). Cells were cultured at 37°C in a humidified atmosphere with 5% CO_2_.

Coxsackievirus A16 (CVA16) and enterovirus 71 (EV71) BrCr strain were obtained from China Center for Type Culture Collection (CCTCC) at Wuhan University. A clinical isolated EV71 Fuyang strain belongs to C4a cluster of the C4 subgenotype as verified through sequence analysis of the VP1 region [11]. EV71 was propagated in RD cells and CVA16 in Vero cells. Virus titers were determined in the corresponding cells by measuring plaque forming units (PFU) of CVA16 and TCID_50_ for EV71 infection [12].

### 2.3. Antibody and Reagents

Antibody against I*κ*B*α* (9242S) was purchased from Cell Signaling (Beverly, MA, USA), and anti-GAPDH monoclonal antibody (MB001) was purchased from Bioworld Technology (Minneapolis, MN, USA). The recombinant human TNF*α* was purchased from Sino Biological Inc. (Beijing, China). HRP-conjugated secondary antibodies and chemical reagents including quercetin, quercitrin, isoquercitrin, acyclovir (ACV), and methylthiazolyldiphenyl-tetrazolium bromide (MTT) were purchased from Sigma-Aldrich (Shanghai, China). The Super Signal ECL reagent kit was purchased from Thermo Fisher (Rockford, IL, USA).

### 2.4. Cytotoxicity Assay

Vero cells (2 × 10^4^ cells) were seeded into 96-well plate and incubated for 12 hr. Varying concentrations of extracts at up to 1 mg/mL were added into the medium. Both treated cells and untreated cells were incubated for 72 hr, followed by incubation with MTT (0.5 mg/mL) for another 4 hr. The ratio of optical density (OD) at 570 nm of treated cells to OD_570_ of untreated cells was expressed as the survival rate of cells. Triplicate wells were analyzed for each concentration. CC_50 _ was defined as the concentration of the extract to reduce the viable cell by 50% relative to the untreated control cells.

### 2.5. Antiviral Assays

EV71 (Fuyang strain and BrCr strain) or CVA16 at a multiplicity of infection (MOI) of 0.2 was used to infect the treated and mock-treated cells. To study the antiviral activity of *Houttuynia cordata* (HCT) and *Mentha haplocalyx* (MHB), Vero cells infected with EV71 or CVA16 were treated with or without a herb extract of various concentrations. The uninfected cells were used as negative control. After 72 hr of infection, cells were stained with crystal violet after fixation of the cells with 3% formaldehyde and captured by a camera and then DMSO was added into the wells to dissolve crystal violet, and the absorbance of 570 nm was measured in a Versa Max microtiter plate reader (Molecular Devices, Sunnyvale, CA, USA). The concentration of an extract that is necessary for 50% inhibition of the virus-induced CPE was expressed as the IC_50_.

To verify the antiviral effect, secondary infection was performed. Serial dilutions of culture supernatants collected from EV71 or CVA16 infected cells were employed to infect monolayers of Vero cells and to determine the titration of infectious viruses as described [13]. The TCID_50_ numbers were calculated using the method of Reed and Muench [12]. The plaque formation was assessed 5 days postinfection (PI) after being fixed with 3% formaldehyde for 20 min and then followed by crystal violet staining as described [14]. The number of plaque was counted manually. The infection was performed in triplicate samples. 

In time-of-drug addition experiment, HCT extract at 40 *μ*g/mL or MHB extract at 200 *μ*g/mL were added at time points specified in the figures. They were either added before viral adsorption, during adsorption (−2 to 0 hr PI), or at varying times after viral adsorption. HCT or MHB was left in the medium throughout the infection assay. The cytopathic effect was determined by measuring cell viability using MTT assay [15] at 72 hr PI. An inhibition rate was calculated as a percentage of (OD_treated_ − OD_infected_) over (OD_uninfected_ − OD_infected_).

To study whether the extracts directly deactivate the virion, EV71 or CVA16 was preincubated with 40 *μ*g/mL HCT and 200 *μ*g/mL MHB in 20 *μ*L, respectively, or remained untreated. The viruses were then used to infect Vero cells cultured with 1 mL culture medium. Alternatively, Vero cells in 24 well plates were preincubated with 40 *μ*g/mL HCT or with 200 *μ*g/mL MHB at 37°C for 2 hr or remained untreated. At the end of the treatment, the extract was removed by replacing the culture medium with fresh DMEM or left in the culture, and the cells were infected with EV71 or CVA16 (MOI was at 0.2, but final concentrations of HCT and MHB were at 0.8 *μ*g/mL and 4 *μ*g/mL, resp.). The samples were harvested at 48 hr PI for titration of infectious viruses with a secondary infection assay.

### 2.6. Immunoblotting Assays

A buffer containing 150 mM NaCl, 50 mM Tris-HCl (pH 7.4), and 1% NP-40 was employed to encourage lysis of cells and to solubilize proteins. Protease and phosphatase are also added to prevent the digestion of the sample by its own enzymes. Prior to protein immobilization on a polyvinylidene difluoride membrane (Millipore), sample proteins were separated using SDS polyacrylamide gel electrophoresis (SDS-PAGE). After blocking, the membrane was incubated with a dilute solution of primary antibody and then with a horseradish peroxidase-linked secondary antibody under gentle agitation. Depending on incubation with a substrate, the images were captured using Alpha Innotech FlourChem-FC2 imaging system and developed manually.

### 2.7. Semiquantification of Proinflammatory Gene Expression Using PCR or qPCR

Vero cells infected with EV71 or CVA16 were treated with HCT or MHB, respectively. Then total RNA was extracted by using TRIzol (Invitrogen, Carlsbad, CA, USA) and following the standard protocol. With a Nanodrop reader, the quantity and purity of total RNA were measured. DNA was synthesized using reverse transcriptase MMLV (RNase H^−^) from 500 ng total RNA with oligo-dT primer. cDNA was amplified by using gene-specific primers and Taq DNA Polymerase (TaKaRa, Tokyo, Japan). 

Real-time PCR was carried out using the SYBR qPCR Mix (TOYOBO, Japan). The protocol was carried out for 40 cycles, comprising 95°C for 1 min, 95°C for 15 s, and 60°C for 30 s. The threshold cycle (Ct) values obtained from these experiments indicated the fractional cycle numbers at which the amount of amplified target reached a fixed threshold. The 2^−ΔΔCt^ method [16] was used to quantify and normalize the relative quantification data. Data were emerged as fold change (2^−ΔΔCt^), which was the amount of IL-6 mRNA, normalized to the internal control (GAPDH). All PCR reactions were performed with equal amounts of cDNA using the Bio-Rad C1000 Real-Time PCR System. A dissociation curve was drawn for each primer pair to assess that there was no primer-dimer formation.

Primer sequences were as follows: IL-6: 5′-AACCTGAACCTTCCAAAGATGG and 5′-TCTGGCTTGTTCCTCACTACT; GAPDH: 5′-ATGACATCAAGAAGGTGGTG and 5′-CATACCAGGAAATGAGCTTG. 

### 2.8. HPLC Profiling and Quantitative Determination of the Chemical Components in the Extract of HCT

The identity of the water extract was characterized by HPLC profiling for several flavonoids. The separations were carried out on a Thermo Scientific Hypersil GOLD column (4.6 mm × 250 mm, 5 *μ*m). The column temperature was set at 25°C. The mobile phase consisted of 0.1% trifluoroacetic acid (A) and acetonitrile (B). The gradient condition was 15% B (V/V) at 0–16 min, 15–25% B at 16–30 min, 25% B at 30–32 min, 25–30% B at 32–35 min, 30% B at 35–37 min, 30–33% B at 37–40 min, 33% B at 40–45 min, 33–60% B at 45–60 min, and 60–15% B at 60–65 min. The flow rate was 0.8 mL/min and the injection volume was 20 *μ*L. Detection wavelength was set at 360 nm. The water extract was identified by comparing their retention times and UV spectra with those of the markers.

## 3. Results

### 3.1. Literature Survey of Plant Materials Used for HFMD

We generated a list of the most commonly used or recommended plant materials for HFMD as listed in Table 1. In general, most of the herbs in the list are bitter or bitter-cold in nature and capable of expelling damp heat in the lung or spleen-stomach, which is the principle for treating diseases of dampness and damp heat. More specifically, the overwhelming majority of the materials have been used for inflammatory and infective diseases. For example,* Lonicera japonica* and pharmaceuticals containing extracts from the flower buds are widely used in China for common cold and for inflammatory diseases of the upper respiratory track. *Isatis indigotica*, *Scutellaria baicalensis*, and *Glycyrrhiza uralensis* were found to have anti-Japanese encephalitis virus, SARS-coronavirus, and respiratory syncytial virus activity [17–20]. Although *Houttuynia cordata* and *Glycyrrhiza uralensis* have been reported with anti-EV71 activity [21, 22], most of the plants have not been evaluated for their activity against HFMD pathogens. The chemical components for the antiviral activity also remain elusive.

### 3.2. Sample Preparation and Antiviral Activity Evaluation

To perform systematic evaluation of the materials for their antiviral and anti-infective activities, herbal materials were purchased from local pharmacy stores and authenticated following methods listed in Chinese Pharmacopeia. The materials were extracted in hot water, and the water extracts were lyophilized. The lyophilized powders were reconstituted in distilled water and tested for cytotoxicity prior to the evaluation for their antiviral activity. At up to 1000 *μ*g/mL concentrations, all extracts except that from *Scutellaria baicalensis* showed no cytotoxic effect in Vero cells. *Scutellaria baicalensis* extract started to show toxic effect at approximately 100 *μ*g/mL in Vero cells with an estimated CC_50_ value of 240.2 *μ*g/mL. To assay for their antiviral activity against EV71 and CVA16 infection, monolayers of Vero cells were treated with an extract at 1000 *μ*g/mL or with *Scutellaria baicalensis* extract at a nontoxic concentration of 30 *μ*g/mL for 2 hr or remained untreated. The cells were then infected with EV71 or CVA16 (MOI = 0.2) for 72 hr. At the end of an infection, the cells were fixed with formaldehyde and examined visually for cytopathic effect. Although recommended or widely used for HFMD disease, only three materials showed moderate to strong activity against EV71 or CVA16 infection (Table 1). The extract of *Houttuynia cordata* significantly blocked the cytopathic effect associated with EV71. The extract did not exhibit significant activity against CVA16 infection. In contrast, MHB treatment significantly blocked the cytopathic effect by CVA16 but not by EV71 infection. An extract from* Glycyrrhiza uralensis* was reported with strong activity against EV71 infection [22]. We found that the extract was marginally active against EV71 and CVA16 infection. Further studies demonstrated that glycyrrhizic acid possessed anti-EV71 activity and anti-CVA16 activity at millimolar concentrations.

### 3.3. Validation and Determination of IC_50_ Values of Plant Materials with Antiviral Activities

**Figure 1:**
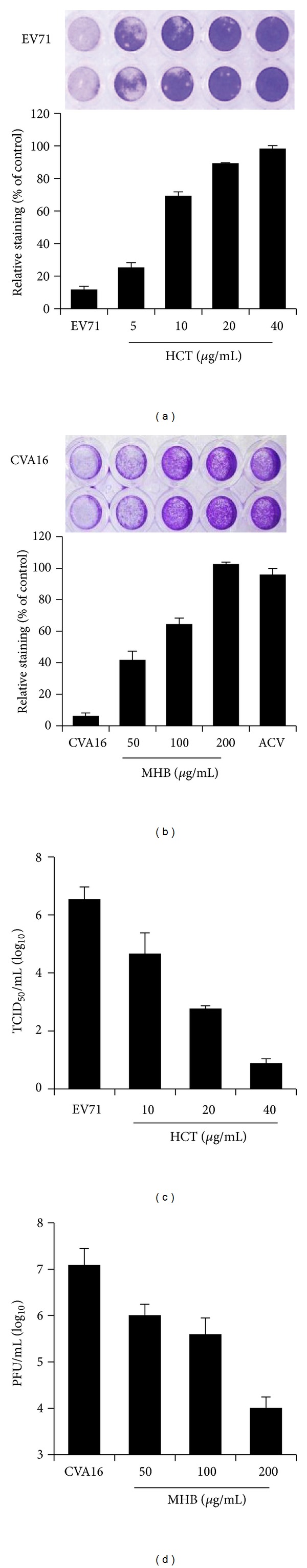
Antiviral activity evaluation of HCT and MHB extracts against EV71 and CVA16. (a, b) Pretreatment of Vero cells with HCT or MHB blocks the cytopathic effect by virus infection. Vero cells were pretreated with or without a herb extract at concentrations as indicated for 2 hr, and the cells were then infected with EV71 (a, Fuyang strain) or CVA16 (b) at an MOI of 0.2 TCID_50_/cell or PFU/cell. After incubation for 72 hr, the cells were fixed with 3% formaldehyde, stained with 0.5% crystal violet. The cells were photographed. To quantitatively measure the staining, crystal violet was extracted by DMSO, and the absorbance at 570 nm was measured colorimetrically. An increase in reading compared with that of an infected control indicates an inhibitory effect of the extract against virus infection. Data are presented as mean ± SE of duplicate samples. (c, d) HCT and MHB treatments reduce infectious virion production of EV71 and CVA16, respectively. Vero cells were infected with EV71 or CVA16 (MOI = 0.2) in the presence or absence of a herbal extract at indicated concentrations. The cells and culture supernatants were harvested at 48 hr PI and determined for virus titration by TCID_50_ assay for EV71 (c) and by plaque forming assay for CVA16 (d). Data are presented as mean ± SE of triplicate samples. The results are representative of two independent experiments.

We then focused our effort on validation of the anti-EV71 activity by HCT and the anti-CVA16 activity of MHB by determining the IC_50_ values. Vero cells were treated with HCT or with MHB at varying concentrations 2 hr prior to inoculation. The cells were then infected with EV71 or CVA16 in the presence or absence of the extracts for 72 hr. As shown in Figures 1(a) and 1(b), pretreatment with the extracts dose dependently blocked the cytopathic effect by those viruses. The IC_50_ value of HCT to block EV71 infection was estimated at 8.9 and 20.6 *μ*g/mL against a Fuyang strain and the BrCr strain of EV71, respectively. The IC_50_ value of MHB to block CVA16 infection was estimated at 70.3 *μ*g/mL, while the IC_50_ for HCT to block CVA16 infection was determined at 186.3 *μ*g/mL. The production of infectious virion was quantitatively determined in parallel experiments to correlate the cytopathic effect with infectious virion production. As shown in Figure 1(c), EV71 production (Fuyang) was reduced by approximately 1.8, 3.7, and 5.6 logs in samples treated with HCT at 10, 20, and 40 *μ*g/mL, respectively. In comparison, MHB at 50, 100, and 200 *μ*g/mL reduced CVA16 production by 1.1, 1.5, and 3.1 logs, respectively (Figure 1(d)).

### 3.4. Time-of-Drug-Addition Study Suggests That HCT Blocks EV71 Infection at an Early Stage of Infection, While MHB Targets Multiple Events in Suppression of CVA16 Infection

**Figure 2:**
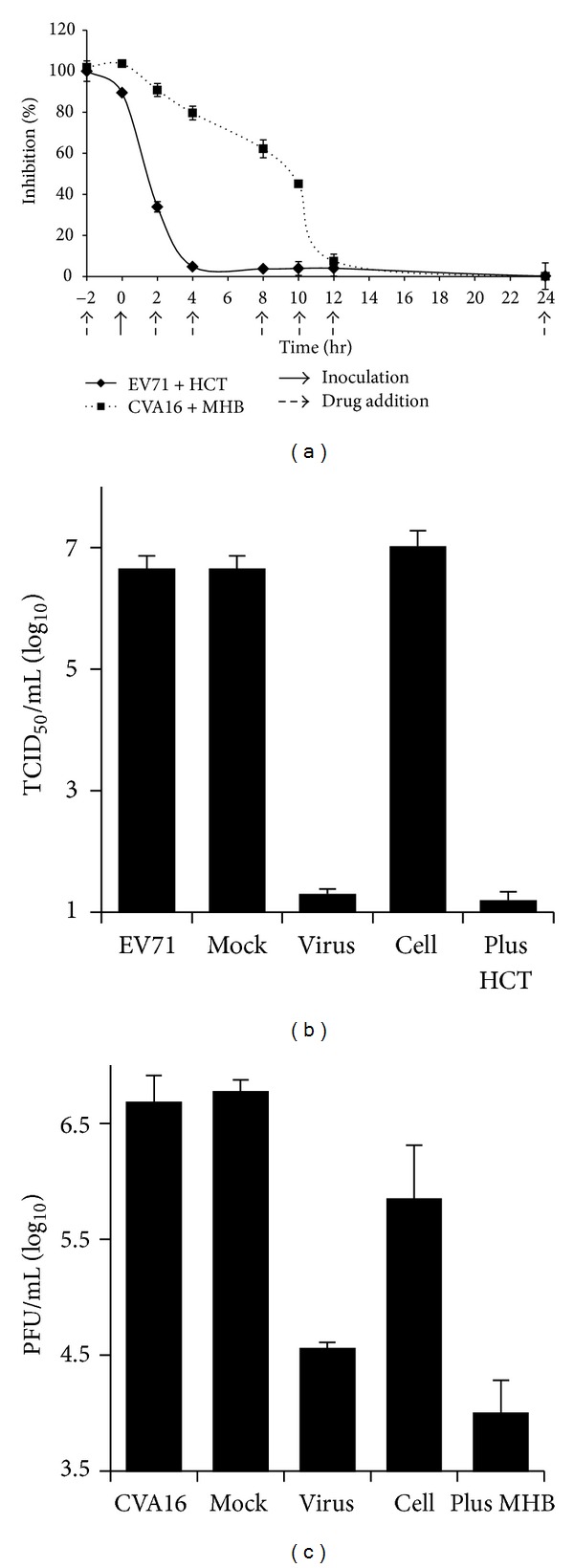
HCT directly inactivates EV71 virion, whereas MHB blocks CVA16 infection by targeting both the virion and cellular factors. (a) Time course study of HCT and MHB antiviral activity. HCT at 40 *μ*g/mL and MHB at 200 *μ*g/mL were added at 2 hr prior to (−2 hr), during (0 hr), or postvirus inoculation at times as indicated (2 hr, 4 hr, 8 hr, 10 hr, 12 hr, and 24 hr PI). Virus infection was determined at 72 hr PI by measuring cell viability colorimetrically. Data are presented as an inhibition rate which is calculated as described in Materials and Methods. (b, c) Coincubation of EV71 with HCT abolishes EV71 infectivity, and MHB inhibits CVA16 infection likely through viral and cellular factors. EV71 (b) or CVA16 (c) in 20 *μ*L culture medium was mock-treated or treated with HCT at a concentration of 40 *μ*g/mL or MHB at 200 *μ*g/mL in a 37°C water bath, respectively, for 2 hr (virus). The treated samples were then added to 1.0 mL fresh culture medium and used to infect Vero cells (final MOIs at 0.2 and final concentrations of HCT and MHB were at 0.8 *μ*g/mL and 4 *μ*g/mL, resp.). In parallel experiments, Vero cells were pretreated with HCT at 40 *μ*g/mL or with MHB at 200 *μ*g/mL at 37°C for 2 hr. The drugs were replaced with fresh culture medium (cell) or left in the culture medium (plus drug). The cells were then infected with EV71 or with CVA16 at equal MOIs. Virus infection was assayed by secondary infection assays at 48 hr PI. Pretreatment of EV71 but not Vero cells with HCT abolished the infectivity of EV71 virus. Treatment of CVA16 or Vero cells with MHB blocked CVA16 infection. Data are presented as mean ± SE of triplicate samples. The results are representative of two independent experiments.

Virus infection is initiated by virus attachment to host cells, followed by virus cell entry and replication intracellularly. To preliminarily determine at what stage of infection the extracts blocked virus infection, monolayers of Vero cells were treated with HCT at 40 *μ*g/mL or MHB at 200 *μ*g/mL 2 hr prior to inoculation or at varying times of virus infection. The effect of drug addition on virus infection was determined at 72 hr PI. As shown in Figure 2(a), addition of HCT prior to the inoculation or during the inoculation significantly suppressed EV71 infection. The effect became less significant when HCT was added at later time points. In comparison, MHB was effective when used prior to or within the first four hours of inoculation. Again, the effect became less significant when added thereafter. Receptor-mediated endocytosis of virus cell entry is generally a rapid process. The result suggests that HCT likely targets EV71 virus by blocking virus cell attachment or by direct inactivation of infectious virion, whereas MHB potentially targets multiple events of CVA16 infection.

To determine whether HCT extract directly deactivates EV71 virion, 1 × 10^4^ TCID_50_ EV71 in 20 *μ*L culture medium was incubated with HCT at 40 *μ*g/mL for 2 hr or remained untreated. The viruses were then used to infect Vero cells at an MOI of 0.2 TICD_50_/cell (the final concentration of HCT was 0.8 *μ*g/mL). In parallel experiments, Vero cells in 24 well plates were preincubated with 40 *μ*g/mL of HCT at 37°C for 2 hr or remained untreated. At the end of the treatment, the extract was removed by replacing the culture medium with fresh DMEM or left in the culture, and the cells were infected with EV71 at 0.2 TICD_50_/cell. As shown in Figure 2(b), pretreatment of EV71, but not the host cells, with HCT abolished the infectivity of EV71 virus, indicating that HCT directly deactivates EV71 virion. In a similar experiment, we found that preincubation of CVA16 or the host cells with MHB reduced infectious CVA16 production by 2.1 logs and 0.9 logs. The result suggests that MHB potentially targets several steps of CVA16 infection (Figure 2(c)).

### 3.5. Antiviral Activity Evaluation of the Flavonoids from HCT

**Figure 3:**
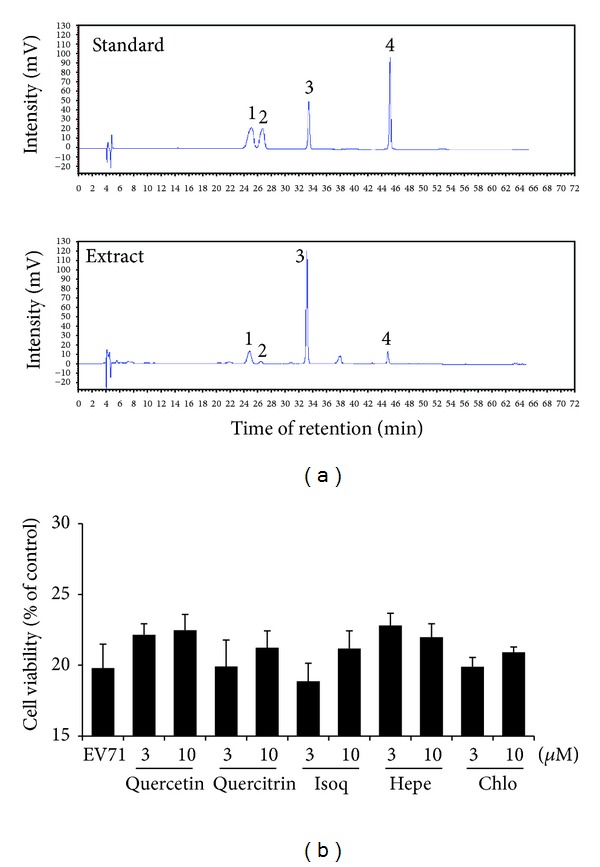
Treatment with flavonoids or chlorogenic acid of HCT does not block EV71 infection. (a) HPLC profiling of water extract of HCT. The identity of the water extract of HCT was characterized by HPLC profiling on a Hypersil GOLD column (4.6 mm × 250 mm, 5 *μ*m) and acetonitrile with increasing concentration of 0.1% trifluoroacetic acid as a mobile phase. Hyperoside (peak 1), isoquercitrin (2), quercitrin (3), and quercetin (4) were used as standards. (b) Flavonoids or chlorogenic acid in HCT has no antiviral effect against EV71 infection. Vero cells were pretreated with or without quercetin, quercitrin, isoquercitrin (Isoq), hyperoside (Hepe), or chlorogenic acid (Chlo) at indicated concentrations. The cells were then infected with EV71 at an MOI of 0.2 TCID_50_/cell for 72 hr, and cell viability was measured colorimetrically. Data are presented as mean ± SE of triplicate samples. The results are representative of two independent experiments.

The chemical contents of HCT and MHB are well characterized. HCT contains 2-undecanone in the essential oil and water soluble chlorogenic acid and flavonoids like quercetin, quercitrin, isoquercitrin, and hyperoside, while the involatile components of MHB include triterpenoids and flavonoids like acacetin, tilianin, and linarin. Several flavonoids like kaempferol [23] and chrysosplenetin [24] have recently been shown with strong antiviral activity against EV71 infection. In a recent study, we also found the flavonoids accounted for the anti-HSV activity of *H. cordata* [25]. To determine whether the flavonoids were responsible for *H. cordata* anti-EV71 activity, flavonoid content in HCT was first profiled by HPLC analysis (Figure 3(a)). The extract contained quercetin, quercitrin, isoquercitrin, and hyperoside. When tested for their antiviral activity against EV71 infection using the flavonoids purchased from a commercial source, the compounds did not display significant activity against EV71 infection (Figure 3(b)).

### 3.6. Anti-Inflammatory Activity of the Herbal Materials Used for HFMD

**Figure 4:**
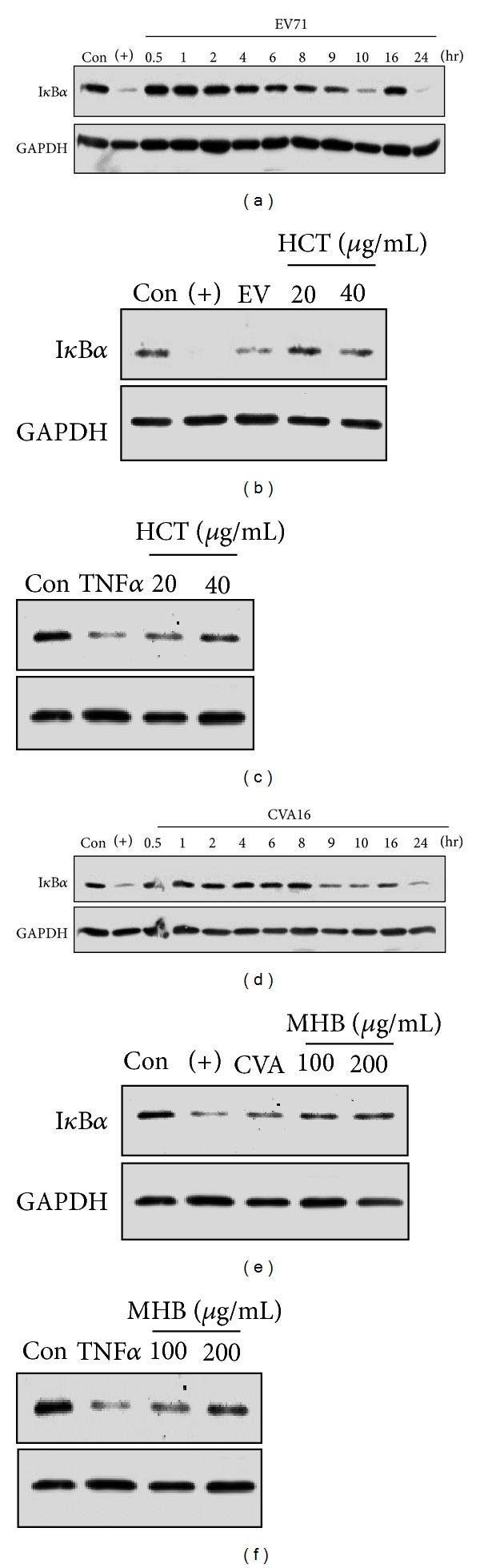
Inhibition of infection-induced I*κ*B*α* degradation by HCT and MHB. (a, d) Determination of the time courses of I*κ*B*α* degradation during EV71 and CVA16 infections. Vero cells were infected with EV71 (a) or CVA16 (d) at an MOI of 3 for indicated times. The cytoplasmic levels of I*κ*B*α* proteins were determined by immunoblotting assay. At 10 hr PI, I*κ*B*α* was significantly degraded implying the NF-*κ*B activation. (b, c) HCT treatment blocks I*κ*B*α* degradation induced by EV71 infection and TNF*α* treatment. Vero cells were infected with EV71 (b, MOI = 3) for 10 hr or treated with 1 ng/mL TNF*α* (c) for 20 min in presence or absence of HCT at indicated concentrations. I*κ*B*α* degradation was detected by immunoblotting assay. (e, f) MHB treatment blocks I*κ*B*α* degradation induced by CVA16 infection and TNF*α* treatment. Vero cells were infected with CVA16 (e, MOI = 3) for 10 hr or treated with 1 ng/mL TNF*α* (f) for 20 min in presence or absence of MHB at indicated concentrations. I*κ*B*α* degradation was detected by immunoblotting assay. GAPDH was used as a loading control. The results are representative of two independent experiments. TNF*α* (+) at 3 ng/mL was used as a positive control for I*κ*B*α* degradation.

Virus infection tends to promote proinflammatory cytokine production, and elevated production of IL-1*β*, IL-6, and IL-8 has been detected in patients with HFMD [26]. The production of IL-6 and IL-8 is regulated transcriptionally by NF-*κ*B pathway, whose activation can be assayed by monitoring the degradation of I*κ*B*α*, the inhibitory protein of NF-*κ*B activity. We next investigated whether the extracts from commonly used herbs for HFMD had activity in suppressing inflammatory response by investigating whether the extracts blocked I*κ*B*α* degradation. As shown in Figure 4, EV71 and CVA16 infection promoted I*κ*B*α* degradation at approximately 10 hr PI (Figures 4(a) and 4(d)). Consistent with the results from antiviral studies, pretreatment of Vero cells with HCT or MHB blocked I*κ*B*α* degradation caused by EV71 and CVA16 infection (Figures 4(b) and 4(e)), respectively. In addition, the extracts also showed inhibitory activity against TNF*α*-induced I*κ*B*α* degradation (Figures 4(c) and 4(f)), indicating that the extracts had activity against proinflammatory factor stimulation.

**Figure 5:**
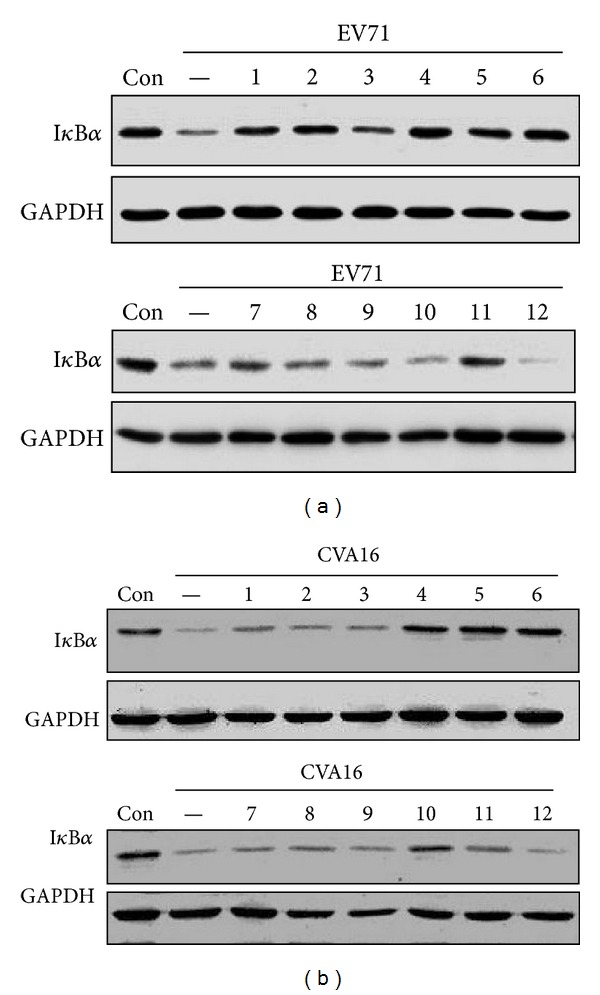
The effect of herb extracts on virus-induced I*κ*B*α* degradation. Vero cells were pretreated with extracts (1: EFT, 2: IIFL, 3: GUF, 4: GJE, 5: MHB, 6: HCT, 7: IIFS, 8: LJT, 9: FSV, 10: ALL, 11: SBG, 12: AAB) for 2 hr and then infected with EV71 (a) or CVA16 (b) at an MOI of 3 for 10 hr. I*κ*B*α* degradation was detected by immunoblotting assay.

To extend those findings, we investigated whether extracts that showed no antiviral activities possessed activity against proinflammatory response to viral infection. To this end, Vero cells were pretreated with the extracts or remained untreated. The cells were then infected with EV71 or CVA16. I*κ*B*α* degradation was detected by immunoblotting analysis. As shown in Figures 5(a) and 5(b), several extracts showed inhibitory activity against infection-induced I*κ*B*α* degradation by EV71 or CVA16 infection, indicating that those extracts have anti-inflammatory activity.

**Figure 6:**
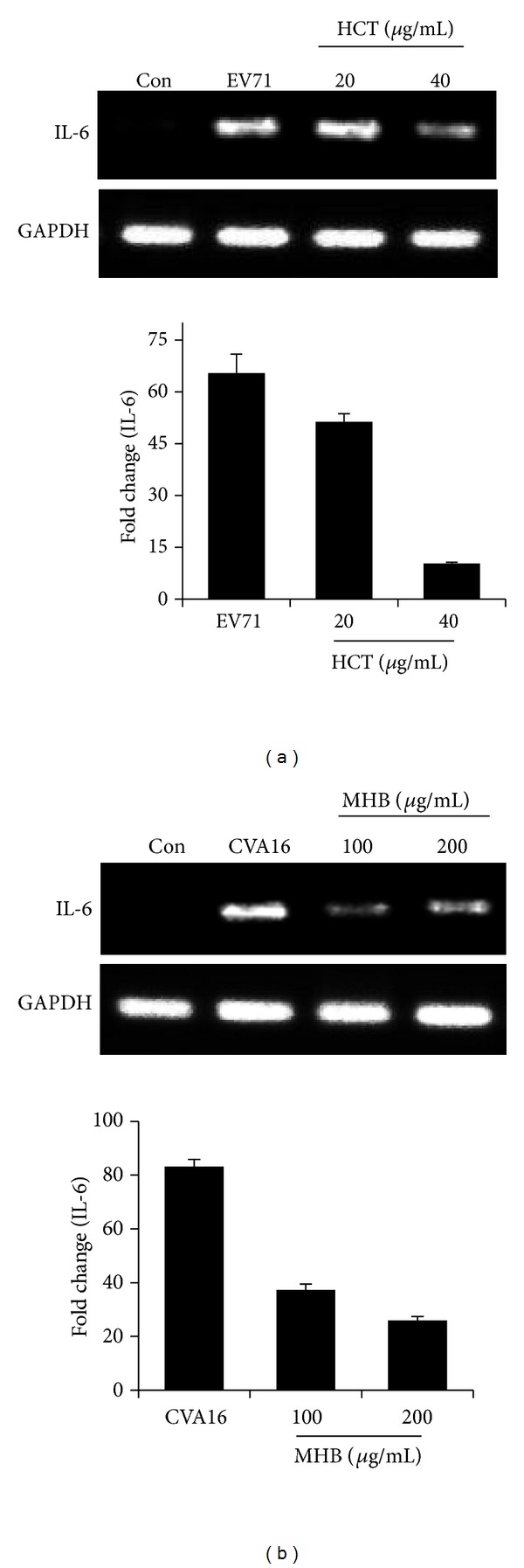
The herb extracts inhibit EV71 or CVA16 infection induced proinflammatory response. Vero cells were infected with EV71 (a) or CVA16 (b) at an MOI of 3 in the presence or absence of HCT or MHB at indicated concentrations. The total RNA was isolated by the TRIzol reagent at 16 hr PI. Five hundred nanograms of total RNA was reverse transcribed and amplified by PCR (upper panels) or by quantitative real-time PCR (lower panels) using primers specific for IL-6 and GAPDH. PCR products were run on a 2.0% agarose gel and visualized with ethidium bromide staining. Data are presented as mean ± SE of triplicate samples. The results are representative of two independent experiments.

**Figure 7:**
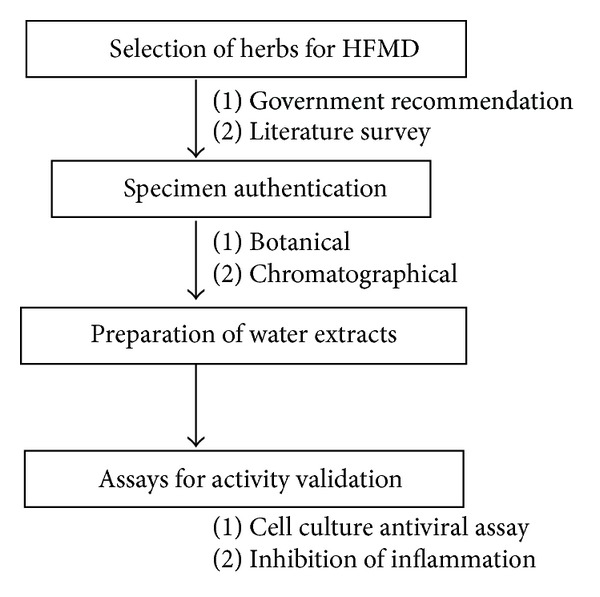
Summary of study design and methodology applied for microbiological validation of medicinal herbs commonly used in China for HFMD.

Finally, the inhibitory effect of HCT and MHB on proinflammatory response was verified by examining expression of downstream genes (Figure 6). The cells were pretreated with HCT or MHB for 2 hr before infection with EV71 or CVA16 at 3 MOI for 16 hr. Both extracts significantly reduced the accumulation of IL-6 mRNA by EV71 and CVA16 infection, respectively, as determined by reverse-transcription PCR (upper panels) as well as quantitative real-time PCR (lower panels).

Together, we showed here that the herbal materials recommended by government agencies or commonly prescribed by practitioners of Chinese medicine possess antiviral activity and/or anti-inflammatory activity, validating the usefulness of those medicinal materials for HFMD disease.

## 4. Discussions

There are currently no treatments for HFMD. Diuretics and intravenous administration of dexamethasone and immunoglobulin-*γ* are among the most commonly recommended drugs for patients with more severe syndromes. Traditional medicine and the use of herbal medicines for HFMD are also among the recommended approaches for the management of HFMD in China. The effectiveness of herbs against HFMD has not previously been evaluated in a laboratory setting. In this study, we selected 12 herbs from government agency-recommended lists and those of the most commonly prescribed. We focused on their antiviral activity evaluation against EV71 and CVA16 and their activity against proinflammatory response since viral infections tend to be associated with proinflammatory response.

EV71 and CVA16 are the two major causative agents causing HFMD. We found that a water extract of *Houttuynia cordata* has strong activity against EV71 infection, while *Mentha haplocalyx* has antiviral activity against CVA16. Although most of the herbs from this list showed no activity against viral infection, several extracts demonstrated activity against proinflammatory response induced by virus infection. The study therefore validates the effectiveness of herbal medicine for an emerging viral disease. 

Many herbs have been commonly prescribed or recommended for the treatment of HFMD; we found *Houttuynia cordata* Thunb. was the only remedy with potent antiviral activity against both EV71 and CVA16. *Houttuynia cordata* has been widely used as an edible vegetable in Chinese and Vietnamese cuisines and has a long history of use in Chinese medicine. The medicinal use of this plant includes its antiseptic, anti-inflammatory, and febrifuge activities. In a previous study, Lin et al. identified *Houttuynia cordata* as the only material with activity against EV71 infection among 22 medicinally used herbs from Taiwan. Consistent with the observation by Lin et al. [21], we also found that the water extract from *Houttuynia cordata* was active against CVA16, extending the antiviral spectrum of this plant. Unlike other widely used herbs, *Houttuynia cordata* was recommended for treatment of HFMD with severe syndrome by the Government of Guangxi Zhuang Autonomous Region, a region with relatively low rate of HFMD incidences and death. Furthermore, the plant contains flavonoids and undecanone. In a preliminary screening study, we failed to detect any antiviral activity with those compounds. Instead, we found that activity was stable against heat and can be extracted out to n-butanol fraction. Further study is warranted to identify the active components from this plant.

It is worthy of noticing that the herbs were tested as single identity, while clinically they are used in combination or formulation containing several other herbals or with cicada molting or even inorganic minerals in some cases. Combined use of those herbs may adversely or inadversely affect the effectiveness of those materials for their antiviral and anti-inflammatory activities. Although this is a laboratory study, the result from this study nevertheless validates the usefulness of herbal medicine for the management of previously undocumented diseases in traditional medicines.

## Figures and Tables

**Table 1:** Evaluation of cytotoxicity and antiviral activity of plant materials used for HFMD in China*.

Latin name	Abbr.	Parts, traditional usage	CC_50_ (*μ*g/mL)	IC_50_ (*μ*g/mL)	
				EV71	CVA16
*Houttuynia cordata* Thunb.	HCT	Stem and leaves, bacterial and viral disease	>1000	8.9 ± 1.6 (FY), 20.6 ± 2.5 (BrCr)	186.3 ± 20.1
*Mentha haplocalyx* Briq.	MHB	Leaves, viral and bacterial disease	>1000	(−)	70.3 ± 4.6
*Glycyrrhiza uralensis* Fisch.	GUF	Stem and root, coughing	>1000	(±)	(±)
*Gardenia jasminoides* Ellis	GJE	Fruits, icteric hepatitis	>1000	(−)	(−)
*Anemarrhena asphodeloides* Bge.	AAB	Stem and root, coughing	>1000	(−)	(−)
*Arctium lappa* L.	ALL	Fruits, coughing	>1000	(−)	(−)
*Eupatorium fortunei* Turcz.	EFT	Stem and leaves, fever	>1000	(−)	(−)
*Lonicera japonica* Thunb.	LJT	Flowers, inflammation	>1000	(−)	(−)
*Scutellaria baicalensis* Georgi	SBG	Root, common cold	240.2 ± 15.4	(−)	(−)
*Isatis indigotica* Fort.	IIFL	Leaves, coughing	>1000	(−)	(−)
*Isatis indigotica* Fort.	IIFS	Root, fever and viral disease	>1000	(−)	(−)
*Forsythia suspensa* (Thunb.) Vahl	FSV	Fruits, viral disease	>1000	(−)	(−)

*The reference of Latin names, abbreviations, parts used in pharmaceuticals and traditional usages of the medicinal plants, and data on antiviral screening against EV71 (Fuyang strain and BrCr strain) and CVA16 are listed. Cytotoxicity was assayed on Vero cells at a maximal concentration of 1000 *μ*g/mL. Those showed toxicity against Vero cells were further tested to determine a CC_50_ value, while those showed no toxic effect at the maximal concentration tested were reported as >1000 *μ*g/mL. IC_50_: 50% inhibitory activity against virus infection-induced cytopathic effect.

Data are presented as mean ± SE of triplicates. All experiments were performed at least twice with triplicate samples. FY: EV71 Fuyang strain; (−): no activity at 1000 *μ*g/mL; (±): marginally active.
